# Legal Cases

**Published:** 1907-03-09

**Authors:** 


### LEGAL CASES.

### INFECTION CAUSED BY NEGLIGENCE OF MEDICAL MAN.

The case of Evans *v.* Mayor of Liverpool (1906, 69 J. P. 263) is an important one as to the liability of a public body for infection caused by the negligence of a medical man employed by them. The defendants, a local authority, acting under the provisions of the Public Health Act, 1875, provided for the uses of the inhabitants of their district a hospital for the reception of persons suffering from infectious diseases. A visiting physician, who was a competent medical practitioner, was appointed to the hospital, and acted under the general directions of the hospitals committee of the defendants, the rules providing (*inter alia*) that he should be responsible for "the treatment of the patients from the beginning to the end of their stay, and also for their freedom from infection when discharged." A son of the plaintiff was treated in the hospital while suffering from a mild attack of scarlet fever, and was ultimately discharged by the visiting physician while still in an infectious condition, and under circumstances which a jury found to amount to a want of reasonable skill and care on his part in and about the discharge; after the boy had returned home he communicated the disease to three other children of the plaintiff. The plaintiff then sued the defendants to recover the expense to which he had been put in regard to the illness of his other children owing to the premature discharge of his son from the hospital by the visiting physician. It was held that the plaintiff was not entitled to recover, for the legal obligation of the defendants extended only to the provision of reasonably skilled and competent medical attendance for the patients, which they had discharged, and that there was no absolute undertaking or obligation on their part that no patient should be discharged by the visiting physician while still in a condition which might cause infection.

At the trial at the Liverpool Assizes the jury found, in answer to specific questions put to them by the learned judge: (1) That there was a want of reasonable skill or care on the part of the visiting physician in or about the discharge of the boy; (2) that in consequence of such want of skill or care the plaintiff suffered the damage complained of; (3) that there was no negligence on the part of the matron or nurses in examining or reporting on the condition of the boy with a view to his discharge; (4) that it was not in consequence of any such last-mentioned negligence that the plaintiff suffered the damage; and (5) that there was an undertaking by the defendants with the plaintiff that their visiting physician should act with reasonable care and skill in and about the discharge of the boy from the hospital. The resolution of the city council appointing the visiting physician provided that the engagement should be subject to the following conditions: "That Dr. A. be responsible for the medical treatment of the patients, and that he visit the Priory Road Hospital every day if requisite; that he sign a book when entering and leaving the hospital; that he make such reports and give such attendance as may be required by the committee; and that he act under the general directions of the committee." From the rules for the directions of officers and servants, which were approved by the Hospitals Committee of the Corporation, it appeared that the infectious diseases hospitals were under the management of that committee, who appointed the visiting physicians, resident medical officers, and matrons, and it was provided that "the visiting physician shall be responsible for the treatment of the patients from the beginning to the end of their stay, and also for their freedom from infection when discharged."

Mr. Justice Walton, in the course of his judgment in this case, said: "It has been contended that the defendants were absolutely bound not to discharge any patient from their hospital who was, in fact, in a condition likely to cause contagion; but it would be a very serious burden upon public bodies who carry on similar hospitals if it were held that they were under any such absolute obligation. There was a great deal of uncontradicted evidence to the effect that, although a patient upon his discharge might appear perfectly well and free from anything that could cause contagion, cases occasionally occurred in which contagion was caused by such a patient; in other words, that it is impossible absolutely to prevent contagion in the case of patients discharged from scarlet fever hospitals. I see no reason for holding that there is an absolute duty imposed on those who have charge of such hospitals not to discharge a patient who might possibly cause contagion to others, a thing which no skill or care can absolutely prevent. I see no reason for holding that there was any such obligation on the defendants." His Lordship therefore gave judgment for the defendants.

An interesting performance is announced to take place at the Imperial Theatre on the afternoon and evening of March 16. The proceeds will be equally divided between the London Hospital and the Diocesan Church Funds. The programme is of more than usual interest, and is certainly of a somewhat different class from that which is affected by the average amateur, as it will consist of a "Dramatised Version," adapted and arranged by Mrs. W. Hadley and Miss Ouless of Part 1, of Bunyan's "Pilgrim's Progress."

